# Validating a Multidimensional Perspective of the Relationship Between Workplace Bullying, Professional Quality of Life, and Turnover Intention of Chinese Novice Nurses

**DOI:** 10.1155/jonm/6653143

**Published:** 2025-05-27

**Authors:** Simeng Dong, Xinshu Shen, Tong Zhao, Rui Zeng, Min Chen

**Affiliations:** ^1^School of Economics and Management, Chongqing University of Posts and Telecommunications, Chongqing, China; ^2^Department of Oncology, Shengjing Hospital of China Medical University, Shenyang, Liaoning, China; ^3^Department Clinical Laboratory, Liaoning Thrombosis Integrated Traditional Chinese and Western Medicine Medical Center, Shenyang, Liaoning, China; ^4^Department of Gerontology, The Sixth People's Hospital of Shenyang, Shenyang, Liaoning, China; ^5^Counseling Center, Chongqing University of Posts and Telecommunications, Chongqing, China

**Keywords:** novice nurses, professional quality of life, turnover intention, workplace bullying

## Abstract

**Background:** The global shortage of nurses is a pressing social issue, and the high mobility of the nursing profession further exacerbates this challenge. Novice nurses' experiences of workplace bullying significantly increase their risk of leaving the profession. Therefore, exploring the mechanisms by which workplace bullying affects novice nurses' turnover intention is important for alleviating the nursing shortage and maintaining the stability of the nursing workforce.

**Method:** This cross-sectional study of 832 novice nurses in northeastern China self-reports assessed workplace bullying, professional quality of life, and turnover intention-related status. The structural equation model was developed to analyze how workplace bullying (including person-related negative, work-related negative, and organizational injustice) affects turnover intention through the mediating role of the professional quality of life (compassion satisfaction, burnout, and secondary traumatic stress).

**Result:** The results indicated that workplace bullying was significantly associated with novice nurses' turnover intention. Person-related negativity, work-related negative, and organizational injustice can influence turnover intentions by decreasing company satisfaction and increasing burnout; furthermore, work-related negativity can increase turnover intention by exacerbating secondary traumatic stress.

**Conclusion:** This study provides new perspectives for understanding novice nurses' psychological reactions and career decisions in a workplace bullying environment and provides empirical support for nursing management practices. The findings highlight the importance of effective intervention strategies to improve the stability of the nursing team, optimize the quality of patient care, and reduce nurse turnover.

## 1. Introduction

Workplace bullying has been classified as a global public health problem by the World Health Organization (WHO) [1]. Workplace bullying manifests itself in three main types of behaviors, including person-related negative behaviors (e.g., intentional ignoring, public shaming, ridiculing, and irrational work scheduling), work-related negative behaviors (e.g., endless blaming and excessive supervision), and organizational-level injustices (e.g., unreasonable workloads) [2]. As a form of systemic violence, it is widespread across multiple industries and fields, including healthcare, manufacturing, service, and higher education institutions [3–6]. However, workplace bullying is particularly prominent in the healthcare industry, especially in nursing [7–10]. Studies have shown that approximately 40% of nursing staff have experienced workplace bullying, and in high-risk departments such as emergency departments and psychiatry, this percentage is even higher at 60% [11, 12].

Novice nurses, as a specific group within the nursing workforce, are at a significantly higher risk of bullying than senior nurses [13, 14]. International studies have found that in Canada, 39% of novice nurses experienced bullying within 1 year of employment and 51% within 2 years of employment [15]. A Korean study of novice nurses revealed that 73.1% experienced at least one verbal abuse, threats of violence, physical violence, bullying, and sexual harassment, with verbal abuse having the highest incidence at 59.6% [16]. Scholars have suggested that lack of professional experience, immaturity, and being at the bottom of the workplace hierarchy may be the main factors contributing to novice nurses being more likely to be targets of bullying [17–20]. In addition, novice nurses are also affected by the changing healthcare environment and increased patient aggression [21]. Prolonged patient interactions, frequent clinical rotations, challenges of new environments, and the need to bond with patients make them more vulnerable to workplace bullying [22].

Bullying in the workplace can cause severe psychological trauma and stress to individuals, triggering somatic and psychological symptoms such as headaches, sleep disorders, depression, anxiety, emotional exhaustion, and posttraumatic stress disorder [23–25]. These symptoms can significantly reduce an individual's job satisfaction, wellbeing, and empathic fulfillment, diminish work motivation, which in turn affects performance and productivity, decrease the quality of professional life, and increase the risk of leaving the job [26–30]. Workplace bullying has been identified as one of the key factors leading to the increase in employees' willingness to leave their jobs [31]. However, the adverse effects of bullying are not only at the behavioral level but also indirectly lead to leaving a job by impairing the professional quality of life in a sustained manner over time [32]. Professional quality of life is of great importance in the nursing field, which not only directly affects nurses' physical and mental health and work performance but also has a profound impact on the quality of patient care, the stability of the nursing team, and the operational efficiency of the entire healthcare system [26]. Professional quality of life refers to the overall wellbeing felt by nursing professionals at work, including positive emotional experiences such as compassion satisfaction, the emotional rewards gained from helping others; negative emotional experiences such as compassion fatigue, that is, burnout, which characterizes emotional exhaustion, and secondary traumatic stress, which is triggered by trauma exposure [33]. Turnover intention, which is closely related to the quality of professional life, is the psychological tendency of an employee to actively seek to leave his or her current job or organization. It is a behavioral antecedent variable of an employee's comprehensive work environment assessment, career development, and organizational support [34].

The Conservation of Resources Theory and the Cognitive Activation Theory of Stress provide a comprehensive theoretical explanatory framework for understanding how workplace bullying affects nurses' professional quality of life through depletion of resources and stress responses, ultimately leading to an increased turnover intention. The Conservation of Resources Theory states that bullying may induce turnover intention through depletion of psychological resources [35]. Individuals tend to acquire and protect their valued resources (such as psychological resources and social support), and defensive exit behaviors may be triggered when these resources are systematically eroded through bullying [36, 37]. For novice nurses, workplace bullying is a stressor that triggers stress by depleting employee resources [38]. Depletion of resources reduces an individual's ability to cope with stress and may trigger a range of negative consequences. Due to a lack of resources, individuals may feel powerless to change the stressor, in which case, leaving often becomes the ultimate response to bullying [39]. On the other hand, the Cognitive Activation Theory of Stress explains how uncontrollable stress activates pathological stress responses [40]. Prolonged exposure to bullying environments, such as harassment and isolation, increases the physical and psychological load on nurses and is accompanied by negative psychological responses such as emotional exhaustion, feelings of helplessness, and anxiety, which trigger secondary traumatic stress [19]. Emotional exhaustion as well as an increase in secondary traumatic stress can further diminish nurses' sense of job fulfillment and compassionate satisfaction, ultimately leading to an increased turnover intention [26, 27, 29, 41].

Previous studies have explored the relationship between workplace bullying and nurses' turnover intention; however, there are still some gaps. First, international studies on workplace bullying in nursing are mainly based on the Western cultural context, and their findings reveal, to a certain extent, the prevalence of bullying, its harms, and related theoretical mechanisms. However, in Chinese organizations with high power distance, where unequal power distribution is generally accepted, bullying may be more prominent in the Chinese nursing industry. Similarly, Confucian culture, which emphasizes hierarchical concepts and a sense of authority, is deeply rooted in Chinese society. In nursing organizations, this cultural context may lead to easier rationalization of bullying by superiors against subordinates [26, 42]. Nurses may remain silent to unreasonable demands or bullying behaviors from superiors due to respect for authority and hierarchy. This cultural context affects nurses' job satisfaction and burnout and may lead to higher turnover rates. Second, most existing studies stay at the macrolevel and lack in-depth analysis of the differentiated mechanisms of action between different types of bullying (e.g., negative personal behaviors and organizational injustice) and willingness to leave the workplace. In addition, there is a lack of clarity about how workplace bullying affects the quality of professional life (compassion satisfaction, burnout, and secondary traumatic stress), which in turn leads to turnover, and there is a lack of research specifically targeting novice nurses, who are at high risk for turnover.

**Figure 1:**
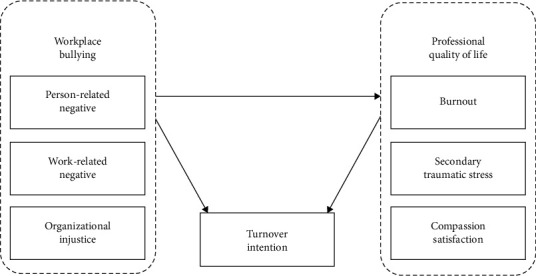
Research framework.

This study focuses on Chinese novice nurses, taking into account the unique cultural context of the Chinese nursing system. Based on the Conservation of Resources Theory and the Cognitive Activation Theory of Stress, from a multidimensional perspective, we hypothesize that the quality of professional life (including compassion satisfaction, burnout, and secondary traumatic stress) mediates the relationship between workplace bullying (including person-related negative, work-related negative, and organizational injustice) and turnover intention of novice nurses. The research framework is shown in Figure 1. This will help to fill the gap of international research on the Chinese nursing context and provide Chinese nursing managers with a theoretical basis and management insights in line with the local reality, which is of great significance to improve the working environment and reduce the turnover rate of Chinese nursing staff.

## 2. Materials and Methods

### 2.1. Participants

This study recruited participants in Northeast China using the snowball sampling method. We set the following inclusion criteria: (1) holding a registered nurse qualification; (2) having participated in the workforce for between 1 and 3 years; and (3) currently working full-time [43].

With the assistance of hospital administrators and nursing staff, we disseminated recruitment information through contact groups and social media platforms. Eligible participants could voluntarily participate in the study through the questionnaire link and were encouraged to share the link with colleagues, friends, and relatives who met the criteria. Participants accessed the online questionnaire link to complete and submit the questionnaire independently. On the front page of the e-questionnaire, we provided a detailed description of the purpose of the study, procedures, and privacy measures. We informed participants of their right to withdraw from the study at any time and ensured that participant confidentiality was strictly maintained throughout the survey.

A total of 848 questionnaires were received, of which 16 were excluded because they did not meet the inclusion criteria. Finally, 832 questionnaires were eligible and included in the analysis. The mean age of the sample was 25.58 ± 1.259 years, and the mean working experience was 2.20 ± 0.662 years. A total of 783 were females (94.110%), and 483 Hukou were urban (58.052%). The top three departments with the highest proportions were internal medicine (150, 18.028%), surgery (142, 17.067%), and obstetrics and gynecology (107, 12.861%).

### 2.2. Measures

#### 2.2.1. Workplace Bullying

This study used the Negative Acts Questionnaire (NAQ) developed by Einarsen and revised by Xun to assess nurses' workplace bullying experiences [2, 44]. The NAQ includes three dimensions and 22 items: person-related negative (9 items), work-related negative (9 items), and organizational injustice (4 items). The scale is rated on a 5-point Likert scale ranging from 1 (never) to 5 (every day), with higher scores indicating greater exposure to workplace bullying. In this study, the overall Cronbach's α of the scale was 0.918, and the Cronbach's α of the three subscales was 0.847, 0.829, and 0.745, respectively, indicating good reliability. The results of the CFA showed that the scale had good validity (*χ*^2^/df = 1.775, CFI = 0.974, TLI = 0.971, GFI = 0.961, and RMSEA = 0.031).

#### 2.2.2. Turnover Intention

The Turnover Intention Scale (TIS) developed by Mobley was used, and the Chinese version was validated [45, 46]. The scale contains four questions. A 5-point Likert scale was used, ranging from 1 (never) to 5 (every day), with a higher total score indicating a higher turnover intention. The consistency reliability of the scale was 0.840, which indicated that the scale had good reliability. The CFA results showed that the scale had good validity (*χ*^2^/df = 1.352, CFI = 0.999, TLI = 0.998, GFI = 0.998, and RMSEA = 0.021).

#### 2.2.3. Professional Quality of Life

The Professional Quality of Life Scale (Pro-QOL) was developed by Stamm, and the Chinese version was revised by Stamm et al. [33, 47, 48]. It consists of three dimensions: compassion satisfaction, burnout, and secondary traumatic stress, and each dimension contains 10 items. The scale is rated on a Likert-5 scale ranging from 1 (never) to 5 (always). In this study, the Cronbach's alpha of the scale was 0.706, and the three subscales had Cronbach's alphas of 0.728, 0.723, and 0.797, respectively, indicating that the scale had good reliability. The CFA results showed that the scale had good validity (*χ*^2^/df = 1.043, CFI = 0.995, TLI = 0.995, GFI = 0.968, and RMSEA = 0.007).

### 2.3. Analysis Process

We used SPSS 24.0 and AMOS 24.0 for data analysis and processing. First, we used SPSS to clear data and generate key variables. Then, we used AMOS software to construct structural equation modeling to calculate path relationships between variables. Finally, we computed 95% confidence intervals (CIs) for the mediating effect using a 5000-times Bootstrap method by writing a user-defined estimand in AMOS [49, 50].

### 2.4. Ethical Considerations

The study was approved by Shengjing Hospital of China Medical University's Ethics Committee (approval number: 2024PS1173K). All procedures followed relevant guidelines and regulations. Each participant signed an informed consent form before completing the questionnaire, and all data were anonymized to ensure the privacy and confidentiality of the participants.

## 3. Result

### 3.1. Preliminary Analysis

Harman's one-way bias analysis showed that the maximum common factor explaining variance extracted from the questionnaire was 21.838%, below the threshold of 40% [51]. Therefore, the data did not suffer from serious common method bias.

Table 1 shows the statistical characteristics and correlation coefficients of the main variables. As shown in Table 1, the turnover intention has significant positive correlations with all dimensions of workplace bullying, including person-related negative (*r* = 0.543, *p* < 0.01), work-related negative (*r* = 0.565, *p* < 0.01), and organizational injustice (*r* = 0.528, *p* < 0.01). Turnover intention also has significant positive correlations with burnout (*r* = 0.434, *p* < 0.01) and secondary trauma stress (*r* = 0.419, *p* < 0.01) but is negatively associated with compassion satisfaction (*r* = −0.362, *p* < 0.01). Finally, significant correlations were found between the dimensions of workplace bullying and the dimensions of professional quality of life.

### 3.2. Structural Equation Modeling Results

**Figure 2:**
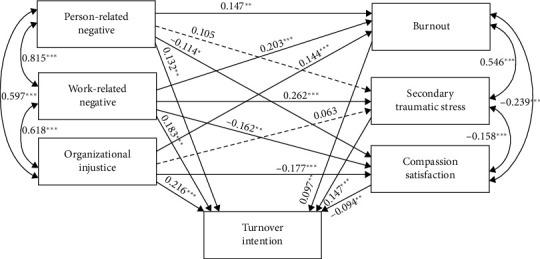
Structural equation model results (standardized estimates). Note: ^∗∗∗^*p* < 0.001, ^∗∗^*p* < 0.01, and ^∗^*p* < 0.05; dashed lines indicate insignificant path coefficients.

A Structural Equation Model (SEM) was developed to examine the relationship between workplace bullying, professional quality of life, and novice nurses' turnover intention, with nurses' gender, age, and domain included as control variables in the model. The overall fit indices of the SEM were *χ*2/df = 1.446, CFI = 0.996, TLI = 0.992, GFI = 0.993, and RMSEA = 0.023, which indicated that the model had a good goodness of fit [52]. The regression results for the structural equation model are shown in Table 2 and Figure 2.

Table 2 shows that person-related negative has a significant positive relationship with burnout (*β* = 0.147, *p* < 0.01) and a significant negative relationship with compassion satisfaction (*β* = −0.114, *p* < 0.05). Work-related negative has a significant positive relationship with burnout (*β* = 0.203, *p* < 0.001) and secondary traumatic stress (*β* = 0.262, *p* < 0.001) and a significant negative relationship with compassion satisfaction (*β* = −0.162, *p* < 0.01). Organizational injustice has a significant positive relationship with burnout (*β* = 0.144, *p* < 0.001) and a significant negative relationship with compassion satisfaction (*β* = −0.177, *p* < 0.001). Also, burnout (*β* = 0.097, *p* < 0.01) and secondary traumatic stress (*β* = 0.147, *p* < 0.001) have a significant positive relationship with turnover intention, while compassion satisfaction (*β* = −0.094, *p* < 0.01) has a significant negative relationship with turnover intention. Finally, person-related negative (*β* = 0.132, *p* < 0.01), work-related negative (*β* = 0.183, *p* < 0.001), and organizational injustice (*β* = 0.216, *p* < 0.001) have a significant positive relationship with turnover intention.

### 3.3. Bootstrap Mediated Effects Test Results

We further tested the effect and significance of each indirect pathway using the 5000-times Bootstrap method, with the effect being significant if the Bootstrap 95% CI did not contain 0. As shown in Table 3, person-related negative can affect turnover intention through burnout (Effect = 0.024 and BootCI = [0.004, 0.058]) and compassion satisfaction (Effect = 0.018 and BootCI = [0.001, 0.048]); work-related negative affects turnover intention through burnout (Effect = 0.029 and BootCI = [0.009, 0.062]), secondary traumatic stress (Effect = 0.058 and BootCI = [0.026, 0.102]), and compassion satisfaction (Effect = 0.023 and BootCI = [0.007, 0.055]); organizational injustice affects turnover intention through burnout (Effect = 0.016 and BootCI = [0.003, 0.035]); and compassion satisfaction(Effect = 0.019, BootCI = [0.007, 0.038]).

## 4. Discussion

Based on Cognitive Activation Theory of Stress and Conservation of Resources Theory, this study constructed a mediation model to provide an in-depth analysis of how workplace bullying (including person-related negative, work-related negative, and organizational injustice) affects novice nurses' turnover intention through three dimensions of quality of professional life (including compassion satisfaction, burnout, and secondary traumatic stress). Findings reveal significant differences in the pathways through which these forms of bullying influence the intention to leave through the quality of professional life. Specifically, person-related negative and organizational injustice affected novice nurses' turnover intention by decreasing compassion satisfaction and increasing burnout. Work-related negative influences turnover intention through all dimensions of quality of professional life, particularly by exacerbating secondary traumatic stress. These findings shed light on the complex mechanisms by which workplace bullying affects novice nurses' quality of professional life and willingness to turnover and provide nursing administrators with a theoretical basis for developing effective interventions.

First, this study confirms that person-related negative in the workplace (e.g., ostracism, ridicule, and devaluation) influence novice nurses' turnover intention through two mediating pathways (increasing burnout and decreasing compassion satisfaction) affecting novice nurses' turnover intention. This finding provides an important mechanistic explanation for understanding the phenomenon of high turnover in the healthcare industry. First, person-related negative significantly impairs nurses' compassion satisfaction, diminishing the positive emotional feedback they receive from patient care and their derived professional self-efficacy [53, 54]. This emotional deprivation directly affects nurses' perception of the meaning of their work, and more critically, it erodes their professional identity as a core psychological resource, resulting in a gradual loss of work ethic among otherwise highly altruistically motivated caregivers. Second, person-related negative exacerbates the classic symptoms of burnout through chronic stress accumulation, including emotional depletion as well as reduced personal fulfillment [55]. This depletion further diminishes nurses' psychological resilience to cope with the demands of their work [56]. This dual-mediation mechanism of action is consistent with resource conservation theory [35, 38], which states that when individuals face persistent resource threats (e.g., workplace rejection), not only do they directly deplete their existing emotional and cognitive resources [57] but they also trigger defensive resource conservation strategies, including emotional alienation and cognitive withdrawal [58]. Ultimately, turnover then becomes an adaptive coping strategy for novice nurses to end further resource loss.

In addition, the results of this study suggest that organizational injustice (including unequal resource allocation, unfair promotion opportunities, and opaque management decisions) significantly enhances novice nurses' turnover intention through a dual mediating path (increasing burnout and decreasing compassion satisfaction). First, organizational injustice directly undermined nurses' compassion satisfaction by weakening organizational trust and psychological contract fulfillment. Specifically, when nurses perceive that their professional contributions are not fairly recognized, they develop a significant sense of organizational injustice. This negative experience significantly inhibits their internal motivation to exhibit caring behaviors by reducing their sense of professional self-efficacy and sense of meaning at work, which, in turn, reduces the positive affective feedback received from patient care [59, 60]. Second, organizational injustice indirectly enhances turnover intention by inducing chronic job stress that leads to burnout. Novice nurses may be more sensitive to such bullying behaviors stemming from their limited career adaptation resources and immature coping strategies [28, 61].

Furthermore, work-related negative influences novice nurses' turnover intention through three dimensions of professional quality of life (increasing burnout and secondary traumatic stress, and decreasing compassion satisfaction), with secondary traumatic stress playing a more prominent role. First, work-related negative (including workplace violence and professional devaluation) significantly reduce nurses' compassion satisfaction by impairing occupational self-efficacy and intrinsic work motivation [62]. This affective deprivation effect makes it difficult for nurses to receive positive feedback from caregiving, creating a give-and-take imbalance [63]. Second, at the same time, work-related negatives manifest in the professional environment as unfair and harmful treatment, which reflects negative social interactions and evaluations in the workplace and triggers psychological and emotional stress in the individual. This leads to job dissatisfaction and burnout, which in turn affect job performance and professional wellbeing [62]. In addition, negative work-related behaviors can weaken an individual's ability to cope with stress, leading to a cumulative effect of stress [64]. As this stress accumulates, secondary traumatic stress increases significantly when individuals feel unable to cope [65]. As a result, they are more likely to consider finding other job opportunities to escape their current negative work environment [66].

Notably, there were selective pathways for the workplace bullying triggering of secondary traumatic stress. Specifically, secondary traumatic stress showed significant mediating effects only in work-related negative of workplace bullying. First, the Conservation of Resources Theory assumes that stressors trigger generalized resource depletion [38]. However, the present study found that a context-specific pattern of resource depletion, including person-related negative and organizational injustice, did not significantly trigger secondary traumatic stress. This discrepancy may stem from the specific nature of nursing, where individuals skip the usual resource assessment process and enter a state of traumatic stress when faced with a direct threat to their physical safety, such as workplace violence [62]. Second, the Cognitive Activation Theory of Stress predicts that stressors uniformly activate cognitive appraisal mechanisms [40]. However, the present study showed that different types of bullying behaviors triggered differential stress responses. Work-related negative (especially violent incidents) were more likely to trigger defensive responses than evaluative coping, possibly due to the immediacy and severity of such threats.

### 4.1. Conclusion

This study delved into the relationship between workplace bullying and novice nurses' turnover intention and the mediating role of professional quality of life. The study found that person-related negative and organizational injustice could increase burnout and decrease compassion satisfaction, ultimately increasing novice nurses' turnover intention. Meanwhile, work-related negative not only affect turnover intention through the mediation of compassion satisfaction and burnout but also affect turnover intention by increasing nurses' secondary traumatic stress.

Based on our findings, we first recommend that hospitals establish a fair work environment that includes equitable resource allocation and transparent promotion mechanisms, strengthen professional ethics and interpersonal relationship training for healthcare workers, and develop and implement effective antibullying policies. Second, mental health support should be provided to nurses, including psychological counseling services and stress management training, and the psychological status of nurses should be continuously monitored and evaluated. Finally, organizational support and leadership should be strengthened, and leadership should demonstrate respect and support for nurses through their behavior and set positive examples.

### 4.2. Limitations

This study has several limitations that need to be addressed in future research. First, the study data were collected through a snowball sampling method, mainly from northeastern China, which limits the generalizability of the findings. Therefore, future studies should expand the sampling area to cover a broader range of regions and healthcare organizations to enhance the representativeness of the findings. Second, because this study used a cross-sectional design, we could only identify associations between variables but not causal relationships. In order to explore causal links between variables, future studies should consider adopting a longitudinal research design. Third, participants may have been biased in their recall of workplace bullying experiences, which may have affected the accuracy of the data. A prospective cohort study design may help reduce recall bias and provide more accurate data. Despite these limitations, the present study makes an important contribution to understanding the impact of workplace bullying on novice nurses' professional quality of life and turnover intention. The findings emphasize the urgency of effective interventions for the problem of workplace bullying and provide valuable foundational data and insights for future research.

## Figures and Tables

**Table 1:** Statistical characteristics and correlation coefficients of the main variables.

Variable	1	2	3	4	5	6	7
1 Turnover intention	1						
2 Person-related negative	0.543^∗∗^	1					
3 Work-related negative	0.565^∗∗^	0.815^∗∗^	1				
4 Organizational injustice	0.528^∗∗^	0.597^∗∗^	0.618^∗∗^	1			
5 Burnout	0.434^∗∗^	0.399^∗∗^	0.412^∗∗^	0.358^∗∗^	1		
6 Secondary trauma stress	0.419^∗∗^	0.356^∗∗^	0.387^∗∗^	0.288^∗∗^	0.622^∗∗^	1	
7 Compassion satisfaction	−0.362^∗∗^	−0.352^∗∗^	−0.365^∗∗^	−0.346^∗∗^	−0.372^∗∗^	−0.287^∗∗^	1
Mean	2.085	1.463	1.668	1.856	2.900	2.763	2.711
SD	0.807	0.481	0.532	0.695	0.609	0.684	0.592

^∗∗^ *p* < 0.01.

**Table 2:** Regression results of structural equation modeling.

Path	*β*	S.E.	t	P
PRN ⟶ BO	0.147	0.070	2.677	0.007
PRN ⟶ STS	0.105	0.080	1.860	0.063
PRN ⟶ CS	−0.114	0.069	−2.042	0.041
WRN ⟶ BO	0.203	0.064	3.621	< 0.001
WRN ⟶ STS	0.262	0.074	4.576	< 0.001
WRN ⟶ CS	−0.162	0.064	−2.835	0.005
OI ⟶ BO	0.144	0.035	3.562	< 0.001
OI ⟶ STS	0.063	0.041	1.521	0.128
OI ⟶ CS	−0.177	0.035	−4.287	< 0.001
PRN ⟶ TI	0.132	0.077	2.858	0.004
WRN ⟶ TI	0.183	0.071	3.845	< 0.001
OI ⟶ TI	0.216	0.040	6.285	< 0.001
BO ⟶ TI	0.097	0.046	2.745	0.006
STS ⟶ TI	0.147	0.039	4.352	< 0.001
CS ⟶ TI	−0.094	0.039	−3.210	0.001
PRN ⟷ WRN	0.815	0.011	18.214	< 0.001
WRN ⟷ OI	0.618	0.015	15.160	< 0.001
OI ⟷ PRN	0.597	0.013	14.781	< 0.001
BO ⟷ CS	−0.239	0.011	−6.694	< 0.001
STS ⟷ BO	0.546	0.014	13.808	< 0.001
CS ⟷ STS	−0.158	0.012	−4.490	< 0.001

*Note:* β-Standardized estimates.

Abbreviations: BO, Burnout; CS, Compassion Satisfaction; OI, Organizational injustice; PRN, Person-related negative; STS, Secondary Trauma Stress; TI, Turnover Intention; WRN, Work-related negative.

**Table 3:** Results of the Bootstrap-mediated effects test.

Indirect path	Effect	S.E.	Bootstrap 95% CI
PRN ⟶ BO ⟶ TI	0.024	0.014	[0.004, 0.058]
PRN ⟶ STS ⟶ TI	0.026	0.015	[0.000, 0.062]
PRN ⟶ CS ⟶ TI	0.018	0.011	[0.001, 0.048]
WRN ⟶ BO ⟶ TI	0.029	0.014	[0.009, 0.062]
WRN ⟶ STS ⟶ TI	0.058	0.019	[0.026, 0.102]
WRN ⟶ CS ⟶ TI	0.023	0.011	[0.007, 0.055]
OI ⟶ BO ⟶ TI	0.016	0.008	[0.003, 0.035]
OI ⟶ STS ⟶ TI	0.011	0.008	[−0.003, 0.027]
OI ⟶ CS ⟶ TI	0.019	0.008	[0.007, 0.038]

Abbreviations: BO, burnout; CS, compassion satisfaction; OI, organizational injustice; PRN, person-related negative; STS, secondary trauma stress; TI, turnover intention; WRN, work-related negative.

## Data Availability

The datasets used and/or analyzed during the current study are available from the corresponding author upon reasonable request.

## References

[1] WHO (2002). *Framework Guidelines for Addressing Workplace Violence in the Health Sector*.

[2] Einarsen S., Hoel H., Notelaers G. (2009). Measuring Exposure to Bullying and Harassment at Work: Validity, Factor Structure and Psychometric Properties of the Negative Acts Questionnaire-Revised. *Work & Stress*.

[3] Arnetz J. E., Fitzpatrick L., Cotten S. R., Jodoin C., Chang C.-H. (2019). Workplace Bullying Among Nurses: Developing a Model for Intervention. *Violence & Victims*.

[4] Hodgins M., McNamara P. M. (2019). An Enlightened Environment? Workplace Bullying and Incivility in Irish Higher Education. *Sage Open*.

[5] Busby L., Patrick L., Gaudine A. (2022). Upwards Workplace Bullying: A Literature Review. *Sage Open*.

[6] Ahmad S., Islam T., D’Cruz P., Noronha E. (2022). Caring for Those in Your Charge: the Role of Servant Leadership and Compassion in Managing Bullying in the Workplace. *International Journal of Conflict Management*.

[7] Magnavita N., Chirico F. (2020). New and Emerging Risk Factors in Occupational Health. *Applied Sciences*.

[8] Mohamed F. B. M., Cheng L. J., Chia X. E. C., Turunen H., He H.-G. (2024). Global Prevalence and Factors Associated with Workplace Violence against Nursing Students: A Systematic Review, Meta-Analysis, and Meta-Regression. *Aggression and Violent Behavior*.

[9] Purpora C., Cooper A., Sharifi C. (2015). The Prevalence of Nurses’ Perceived Exposure to Workplace Bullying and its Effect on Nurse, Patient, Organization and Nursing-Related Outcomes in Clinical Settings: A Quantitative Systematic Review Protocol. *JBI Database of Systematic Reviews and Implementation Reports*.

[10] Chaudhary A., Islam T. (2022). How Workplace Bullying Affects Knowledge Hiding? the Roles of Psychological Contract Breach and Learning Goal Orientation. *VINE Journal of Information and Knowledge Management Systems*.

[11] Trépanier S. G., Fernet C., Austin S. (2015). A Longitudinal Investigation of Workplace Bullying, Basic Need Satisfaction, and Employee Functioning. *Journal of Occupational Health Psychology*.

[12] Magnavita N., Heponiemi T. (2012). Violence towards Health Care Workers in a Public Health Care Facility in Italy: A Repeated Cross-Sectional Study. *BMC Health Services Research*.

[13] Cooper B., Curzio J. (2012). Peer Bullying in a Pre-Registration Student Nursing Population. *Nurse Education Today*.

[14] Dong S., Shen X., Zhao T., Zeng R., Chen M. (2025). Workplace Violence, Psychopathological Symptoms, and Deviant Workplace Behavior Among Nursing Interns in China: A Network Analysis. *BMC Nursing*.

[15] Laschinger H. K. S., Fida R. (2013). A Time-Lagged Analysis of the Effect of Authentic Leadership on Workplace Bullying, Burnout, and Occupational Turnover Intentions. *European Journal of Work & Organizational Psychology*.

[16] Chang H. E., Cho S.-H. (2016). Workplace Violence and Job Outcomes of Newly Licensed Nurses. *Asian Nursing Research*.

[17] Edmonson C., Zelonka C. (2019). Our Own Worst Enemies. *Nursing Administration Quarterly*.

[18] Gamble Blakey A., Smith-Han K., Anderson L., Collins E., Berryman E., Wilkinson T. J. (2019). Interventions Addressing Student Bullying in the Clinical Workplace: A Narrative Review. *BMC Medical Education*.

[19] Peng J., Luo H., Ma Q. (2021). Association between Workplace Bullying and Nurses’ Professional Quality of Life: The Mediating Role of Resilience. *Journal of Nursing Management*.

[20] Vessey J. A., Demarco R., Difazio R. (2010). Bullying, Harassment, and Horizontal Violence in the Nursing Workforce the State of the Science. *Annual Review of Nursing Research*.

[21] Bowllan N. M. (2015). Nursing Students’ Experience of Bullying. *Nurse Educator*.

[22] Magnavita N., Heponiemi T. (2011). Workplace Violence Against Nursing Students and Nurses: An Italian Experience. *Journal of Nursing Scholarship*.

[23] Chakraborty S., Mashreky S. R., Dalal K. (2022). Violence against Physicians and Nurses: A Systematic Literature Review. *Journal of Public Health*.

[24] Islam T., Ahmed I., Ali G. (2019). Effects of Ethical Leadership on Bullying and Voice Behavior Among Nurses. *Leadership in Health Services*.

[25] Karatuna I., Jönsson S., Muhonen T. (2020). Workplace Bullying in the Nursing Profession: A Cross-Cultural Scoping Review. *International Journal of Nursing Studies*.

[26] Jiao R., Li J., Cheng N., Liu X., Tan Y. (2023). The Mediating Role of Coping Styles between Nurses’ Workplace Bullying and Professional Quality of Life. *BMC Nursing*.

[27] Choi S.-H., Lee H. (2017). Workplace Violence Against Nurses in Korea and Its Impact on Professional Quality of Life and Turnover Intention. *Journal of Nursing Management*.

[28] Itzhaki M., Bluvstein I., Peles Bortz A. (2018). Mental Health Nurse’s Exposure to Workplace Violence Leads to Job Stress, Which Leads to Reduced Professional Quality of Life. *Frontiers in Psychiatry*.

[29] Boudrias V., Trépanier S. G., Salin D. (2021). A Systematic Review of Research on the Longitudinal Consequences of Workplace Bullying and the Mechanisms Involved. *Aggression and Violent Behavior*.

[30] Islam T., Chaudhary A. (2022). Impact of Workplace Bullying on Knowledge Hiding: The Mediating Role of Emotional Exhaustion and Moderating Role of Workplace Friendship. *Kybernetes*.

[31] Sauer P. A., McCoy T. P. (2016). Nurse Bullying: Impact on Nurses’ Health. *Western Journal of Nursing Research*.

[32] Yuan T., Ren H., Liang L. (2024). Professional Quality of Life Profiles and Its Associations With Turnover Intention and Life Satisfaction Among Nurses: A Prospective Longitudinal Study. *BMC Psychology*.

[33] Stamm B. H. (2005). *The Professional Quality of Life Scale: Compassion Satisfaction, Burnout, and Compassion Fatigue/Secondary Trauma Scale*.

[34] Mobley W. H. (1977). Intermediate Linkages in the Relationship between Job Satisfaction and Employee Turnover. *Journal of Applied Psychology*.

[35] Hobfoll S. E. (2001). The Influence of Culture, Community, and the Nested‐Self in the Stress Process: Advancing Conservation of Resources Theory. *Applied Psychology*.

[36] Hobfoll S. E., Halbesleben J., Neveu J.-P., Westman M. (2018). Conservation of Resources in the Organizational Context: The Reality of Resources and Their Consequences. *Annual Review of Organizational Psychology and Organizational Behavior*.

[37] Nauman S., Malik S. Z., Jalil F. (2019). How Workplace Bullying Jeopardizes Employees’ Life Satisfaction: The Roles of Job Anxiety and Insomnia. *Frontiers in Psychology*.

[38] Hobfoll S. E. (1989). Conservation of Resources. A New Attempt at Conceptualizing Stress. *American Psychologist*.

[39] Laeeque S. H., Bilal A., Babar S., Khan Z., Ul Rahman S. (2017). How Patient-Perpetrated Workplace Violence Leads to Turnover Intention Among Nurses: The Mediating Mechanism of Occupational Stress and Burnout. *Journal of Aggression, Maltreatment & Trauma*.

[40] Ursin H., Eriksen H. R. (2004). The Cognitive Activation Theory of Stress. *Psychoneuroendocrinology*.

[41] Austin C. L., Saylor R., Finley P. J. (2017). Moral Distress in Physicians and Nurses: Impact on Professional Quality of Life and Turnover. *Psychological Trauma: Theory, Research, Practice, and Policy*.

[42] Wu Y., Buljac-Samardzic M., Zhao D., Ahaus C. T. B. (2024). The Importance and Feasibility of Hospital Interventions to Prevent and Manage Patient Aggression and Violence against Physicians in China: A Delphi Study. *Human Resources for Health*.

[43] Zhang S., Ma C., Meng D. (2018). Impact of Workplace Incivility in Hospitals on the Work Ability, Career Expectations and Job Performance of Chinese Nurses: A Cross-Sectional Survey. *BMJ Open*.

[44] Xun H., Liu H., Tian Z. (2012). A Preliminary Reliability and Validity Study of Chinese Version of the Negative Acts Questionnaire Revised. *Chinese Nursing Management*.

[45] Mobley W. H., Horner S. O., Hollingsworth A. T. (1978). An Evaluation of Precursors of Hospital Employee Turnover. *Journal of Applied Psychology*.

[46] Lu Y., Hu X.-M., Huang X.-L. (2017). The Relationship Between Job Satisfaction, Work Stress, Work–Family Conflict, and Turnover Intention Among Physicians in Guangdong, China: a Cross-Sectional Study. *BMJ Open*.

[47] Stamm B. H. (2013). Measuring Compassion Satisfaction as Well as Fatigue: Developmental History of the Compassion Satisfaction and Fatigue Test. *Treating Compassion Fatigue*.

[48] Zheng X., Yang M., Gao W., Chen F. (2013). The Chinese Version Professional Quality of Life Scale: Testing of Reliability and Validity in Nurses. *Journal of Nursing Science*.

[49] Hayes A. F. (2013). *Introduction to Mediation, Moderation, and Conditional Process Analysis: A Regression-Based Approach*.

[50] Collier J. (2020). *Applied Structural Equation Modeling Using AMOS: Basic to Advanced Techniques*.

[51] Podsakoff P. M., MacKenzie S. B., Lee J.-Y., Podsakoff N. P. (2003). Common Method Biases in Behavioral Research: A Critical Review of the Literature and Recommended Remedies. *Journal of Applied Psychology*.

[52] Fornell C., Larcker D. F. (1981). Evaluating Structural Equation Models with Unobservable Variables and Measurement Error. *Journal of Marketing Research*.

[53] Craigie M., Osseiran-Moisson R., Hemsworth D. (2016). The Influence of Trait-Negative Affect and Compassion Satisfaction on Compassion Fatigue in Australian Nurses. *Psychological Trauma: Theory, Research, Practice, and Policy*.

[54] Rosander M., Salin D., Blomberg S. (2022). The Last Resort: Workplace Bullying and the Consequences of Changing Jobs. *Scandinavian Journal of Psychology*.

[55] Maslach C., Schaufeli W. B., Leiter M. P. (2001). Job Burnout. *Annual Review of Psychology*.

[56] Bakker A. B., Demerouti E. (2017). Job Demands–Resources Theory: Taking Stock and Looking Forward. *Journal of Occupational Health Psychology*.

[57] Grandey A. A., Gabriel A. S. (2015). Emotional Labor at a Crossroads: Where Do We Go From Here?. *Annual Review of Organizational Psychology and Organizational Behavior*.

[58] Diefendorff J. M., Erickson R. J., Grandey A. A., Dahling J. J. (2011). Emotional Display Rules as Work Unit Norms: A Multilevel Analysis of Emotional Labor Among Nurses. *Journal of Occupational Health Psychology*.

[59] Laschinger H. K. S. (2014). Impact of Workplace Mistreatment on Patient Safety Risk and Nurse-Assessed Patient Outcomes. *The Journal of Nursing Administration: The Journal of Nursing Administration*.

[60] Dall’Ora C., Ball J., Reinius M., Griffiths P. (2020). Burnout in Nursing: A Theoretical Review. *Human Resources for Health*.

[61] Waqas A., Haider S., Ahmed R., Khaliq A. A., Selem K. M. (2022). Workplace Violence and Interpersonal Deviance Among Pakistani Nurses: Role of Sense of Coherence. *Current Psychology*.

[62] Nielsen M. B., Einarsen S. (2012). Outcomes of Exposure to Workplace Bullying: A Meta-Analytic Review. *Work & Stress*.

[63] Siegrist J. (1996). Adverse Health Effects of High-Effort/Low-Reward Conditions. *Journal of Occupational Health Psychology*.

[64] de Boer J., Lok A., van’t Verlaat E., Duivenvoorden H. J., Bakker A. B., Smit B. J. (2011). Work-Related Critical Incidents in Hospital-Based Health Care Providers and the Risk of Post-Traumatic Stress Symptoms, Anxiety, and Depression: A Meta-Analysis. *Social Science & Medicine*.

[65] Berry P., Gillespie G., Fisher B., Gormley D., Haynes J. (2016). Psychological Distress and Workplace Bullying Among Registered Nurses. *Online Journal of Issues in Nursing*.

[66] Kim Y., Lee E., Lee H. (2020). Correction: Association Between Workplace Bullying and Burnout, Professional Quality of Life, and Turnover Intention Among Clinical Nurses. *PLoS One*.

